# Reliability of Pressure Pain Threshold (PPT) and Conditioned Pain Modulation (CPM) in Participants with and without Chronic Shoulder Pain

**DOI:** 10.3390/healthcare12171734

**Published:** 2024-08-31

**Authors:** Paraskevi Bilika, Panagiotis Kalamatas-Mavrikas, Nikolaos Vasilis, Nikolaos Strimpakos, Eleni Kapreli

**Affiliations:** 1 Clinical Exercise Physiology & Rehabilitation Research Laboratory, Physiotherapy Department, Faculty of Health Sciences, University of Thessaly, 351 32 Lamia, Greece; mkalamatas@uth.gr (P.K.-M.); nvasilis@uth.gr (N.V.); ekapreli@uth.gr (E.K.); 2 Go Physio Laboratory, Sports Medicine & Rehabilitation Centre, 106 75 Athens, Greece; 3 Health Assessment and Quality of Life Research Laboratory, Physiotherapy Department, Faculty of Health Sciences, University of Thessaly, 351 32 Lamia, Greece; nikstrimp@uth.gr; 4 Division of Musculoskeletal & Dermatological Sciences, Honorary Research Associate, University of Manchester, Manchester M13 9PL, UK

**Keywords:** conditioned pain modulation, pressure pain threshold, reliability, shoulder

## Abstract

The objectives of this study were to estimate the intra-rater and inter-rater reliability of the Pressure Pain Threshold (PPT) and Conditioned Pain Modulation (CPM) in healthy participants and patients with chronic shoulder pain. Additionally, the Standard Error of Measurement (SEM) and Smallest Detectable Change (SDC) were calculated. Thirty-one healthy volunteers and twenty patients with chronic shoulder pain were assessed using the PPT and CPM by two raters, with a 24 h interval between sessions. Excellent intra-rater reliability was demonstrated for PPT, with similar SEM and SDC when assessed by the same rater. The inter-rater reliability for PPTs in patients was moderate to good (ICC = 0.59–0.89) with higher SEM (73.83–121.98 kPa) and SDC (61.58–97.59) values than the asymptomatic group (ICC = 0.92–0.96, SEM = 49.61–103.12 kPa, SDC = 42.01–56.30) respectively. CPM’s intra-rater reliability was good (ICC = 0.82) in the patients and moderate (ICC = 0.67) in the asymptomatic group, while inter-rater reliability was low for the asymptomatic group (ICC = 0.37) and extremely low (ICC = 0.074) for the patients, with comparable SEM and SDC outcomes in both groups. PPT and CPM measurements are highly reliable when conducted by the same rater on the same day. Patients had lower inter-rater PPT reliability but better intra-rater CPM reliability. Clinicians need to be mindful of potential variability when interpreting these test results.

## 1. Introduction

Clinical studies increasingly support the occurrence of central sensitization (CS) in a subgroup of patients with chronic shoulder pain [1]. This subgroup presents with generalized mechanical hyperalgesia, widespread referred pain [2] while appearing to have increased levels of psychological distress such as anxiety, depression, and kinesiophobia [3,4]. CS involves the increased response to the input of stimuli (noxious and potentially noxious) and their altered processing centrally as a result of central nervous system (CNS) sensitization [5]. There is strong evidence that the consolidation of this state may play a role in the long-term maintenance of pain [6] and the development of a type of pain called nociplastic pain [7,8]. 

The clinical manifestation of altered afferent information as well as a differential response from endogenous pain inhibition systems can be assessed with experimentally induced pain tests. Pain Pressure Threshold (PPT) is the most common static test used to assess pain sensitivity. PPT is a simple test in which a stimulus, usually mechanical (or thermal), with gradually increasing intensity is applied to the subject’s skin and the subject is asked to indicate the time point at which the stimulus begins to change from pressure to pain. By assessing PPT in the region of referred pain or at remote uninvolved sites, health professionals can gain information about the predominance of peripheral (sensitization of Ad and C fibers) or CS (general sensitivity due to increased excitability of neurons of the dorsal horn) mechanisms [9,10]. 

It is not yet clear whether static or dynamic tests are a manifestation of CS. Conditioned Pain Modulation (CPM) has been described as a “dynamic inhibitory measure” [10] as it has been considered that this test estimates the ability of the nervous system to intercept the sensation of pain when needed [11]. During CPM, a painful stimulus applied to a remote body region (conditioning stimulus) inhibits the pain response to another stimulus (test stimulus). Dysfunction of descending pain inhibitory systems is thought to contribute to the maintenance of CS and thus to the development of chronic pain disorders [12,13,14]. Indeed, findings of impaired modulation have been observed in patients with chronic pain highlighting the dysfunction of endogenous pain regulation mechanisms in this population [15]. Likewise, CPM has been associated with the clinical response to manual therapy treatment in patients with epicondylalgia [16], the effectiveness of analgesic therapy in patients with knee osteoarthritis [17], and patients with low back pain [18], and with other factors such as psychological distress [19].

It is reported that tests such as PPT and CPM could be a cost-effective and clinically useful component of pain assessment, which will contribute significantly towards the goal of individualized mechanism-based pain management [20]. Both measures have been used in numerous studies to assess patients with shoulder pain [3,21,22,23,24,25,26,27,28,29,30]. However, the absence of appropriate and standardized protocols in patients with shoulder pain makes it difficult to develop normative data [31] and estimate the prevalence of CS in this population [10]. 

Regarding the PPT, one study [30] evaluated the intra-rater and inter-rater reliability of PPT in patients with Subacromial Impingement Syndrome and healthy participants and estimated the SEM and minimum clinical change in this population. However, methodological deficits regarding patient stability and similarity of measurement conditions were observed [32]. On the other hand, few studies have tested the reliability of the CPM [28,33,34,35] and only one study [28] included patients with shoulder pain. However, inter-rater reliability, measurement error and SDC, which are necessary for the application of the test in clinical practice, were not examined.

This study aims to assess the intra-rater and inter-rater reliability of PPT and CPM, and, for the first time, calculate the SEM and SDC in both healthy participants and patients with chronic shoulder pain.

## 2. Materials and Methods

### 2.1. Study Design

The present study was conducted between October 2021 and July 2022 and has received approval from the Ethics Committee of the Physiotherapy Department of the University of Thessaly (No 711/23-09-21). The authors have followed the Guidelines for Reporting Reliability and Agreement Studies (GRAAS) [36]. The measurements took place at the Clinical Exercise Physiology and Rehabilitation Research laboratory of the University of Thessaly and in a private physiotherapy clinic. 

### 2.2. Participants 

The sample was recruited from the University of Thessaly (staff and students), the physiotherapy clinic, and physical therapy clinics in Lamia, via voluntary response sampling. Volunteers responded to a public announcement to recruit participants. Symptomatic participants were adults (18–65 years) with a history of shoulder pain (pain duration at least 3 months and pain intensity ≥3/10 on the Visual Analogue Scale (VAS)). The asymptomatic group included adults (18–65 years) without pain (acute or chronic) at least 3 months before the experimental sessions.

Volunteers were excluded if they had a BMI score above 35 or a neurological, cardiovascular, mental, or systemic disorder. Patients with a BMI above 35 were excluded for two primary reasons: first, to reduce variations in palpation that could arise due to the increased adipose tissue in individuals with higher BMI; second, to minimize variations in pain perception, as previous studies have found differences in obese groups compared to individuals with a healthy weight. Additionally, participants with higher BMI were excluded in similar studies to ensure more consistent and reliable results [30]. Furthermore, they were excluded if they presented with the following: (1) pregnancy, (2) recent surgery, or (3) traumatic injury in the shoulder or neck. All the participants were instructed to maintain their everyday lifestyle, but they were asked to refrain from physical activity and consumption of caffeine, alcohol, nicotine, or painkillers in the last 24 h before the measurements. Moreover, the women evaluated were not menstruating. Women were excluded during their menstruating period based on studies demonstrating an association between the menstrual cycle and variations in pain sensitivity [37,38]. The screening was carried out via phone. The participant’s compliance with the instructions was confirmed verbally upon arrival at the laboratory.

In reliability studies, two main approaches can be used for sample size calculation: estimation and hypothesis testing [39]. Given that our primary goal was to estimate the Intraclass Correlation Coefficient (ICC) with sufficient precision, we opted for the estimation method [39]. Specifically, we aimed to estimate an ICC of 0.90, which is generally considered to represent excellent reliability. To ensure that our estimate was precise, we determined the width of the confidence interval to be 0.15. To achieve this level of precision with an 80% probability (power), the required sample size was calculated to be 19 participants [39]. This calculation was based on the inclusion of 3 repeated measures. This approach ensures that our sample size is adequate to provide a reliable estimate of the ICC, thereby supporting the validity of our study’s findings. For two raters, we aimed to estimate an ICC of 0.90, which is indicative of excellent reliability. To ensure the estimate was precise, we set the width of the confidence interval to 0.20. To achieve this level of precision with an 80% probability (power), the required sample size was calculated to be 25 participants [39]. 

### 2.3. Raters

Two independent raters (PKM and NV), final-year postgraduate students who were trained to use the algometer, performed all the measurements. During the training, the raters learned to apply a constant rate (40 kPa/s) to the algometer, and they became familiar with the process. The raters applied pressure to a stable and solid object for the training and then to the skin of 5 volunteers who did not participate in the analysis. Likewise, the raters were trained to use the same grips and body positions during the experimental sessions. The entire procedure was videotaped with the permission of the participants and used for continuous training of the raters before each measurement day. 

### 2.4. Pressure Pain Threshold

**Figure 1:**
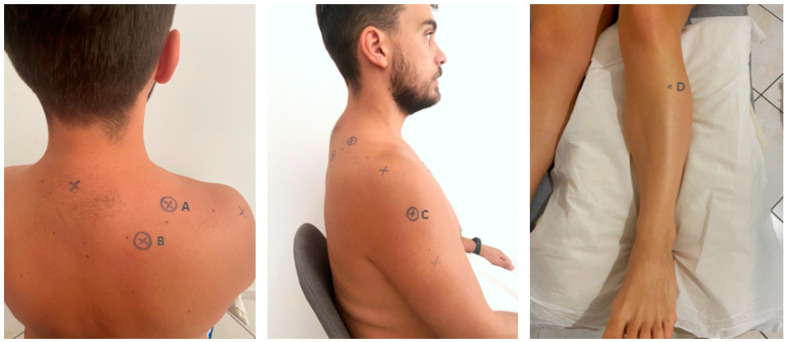
The testing sites: (A) upper trapezius; (B) levator scapulae; (C) middle deltoid; (D) tibialis anterior.

A pressure algometer (Somedic AB, Farsta, Sweden) was used for the measurements. PPT was measured over 3 sites on the shoulder area and 1 distant point randomly using the website https://www.random.org/ accessed on 15 March 2022 (Figure 1). The testing sites were located (A) at the affected or dominant side in the upper trapezius (in the middle of the distance between the C7 spinous process and the acromion [30,40], (B) the levator scapulae (2 cm above the lower insertion of levator scapulae located in the upper medial border of the scapula) [30], (C) the middle deltoid (in the middle of the distance between the acromion and the deltoid trochanter) [40], and the opposite side 5 cm distal to tuberositas tibia in the belly (the thickest portion of the muscle) of tibialis anterior muscle [41] (Figure 1).

**Figure 2:**
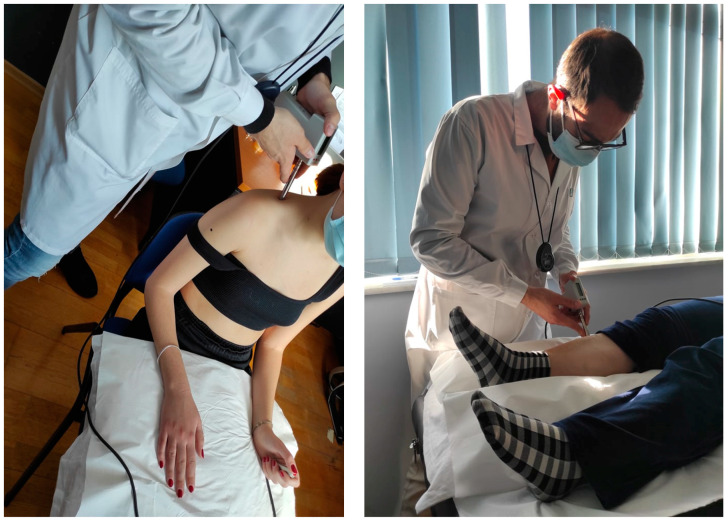
Pressure Pain Threshold testing in the seated and supine position.

The algometer provides visual feedback for the correct pressure rate application (40 kPa/s). For the measurements performed on the shoulder, participants were seated in a chair. Their back was supported, and their forearms rested on a pillow over their legs (Figure 2). To assess the PPT on the tibialis anterior muscle, the subjects lay supine on a medical examination bench with a pillow placed under the testing leg and another placed under the head (Figure 2).

The test was repeated 3 times alternatively, to avoid summing up the pain, with a time interval of 5 min between cycles. Subjects were instructed to press the button when the pressure applied to their skin became unpleasant. The two raters were trained to provide the same instructions and in the same way to patients. The instructions were created according to previously published guidelines [42]. The maximum pressure, before the examinee pressed the button, was recorded. Each rater was blind to the other rater’s assessment and each patient’s self-reported outcome measure scores. 

### 2.5. Conditioned Pain Modulation 

**Figure 3:**
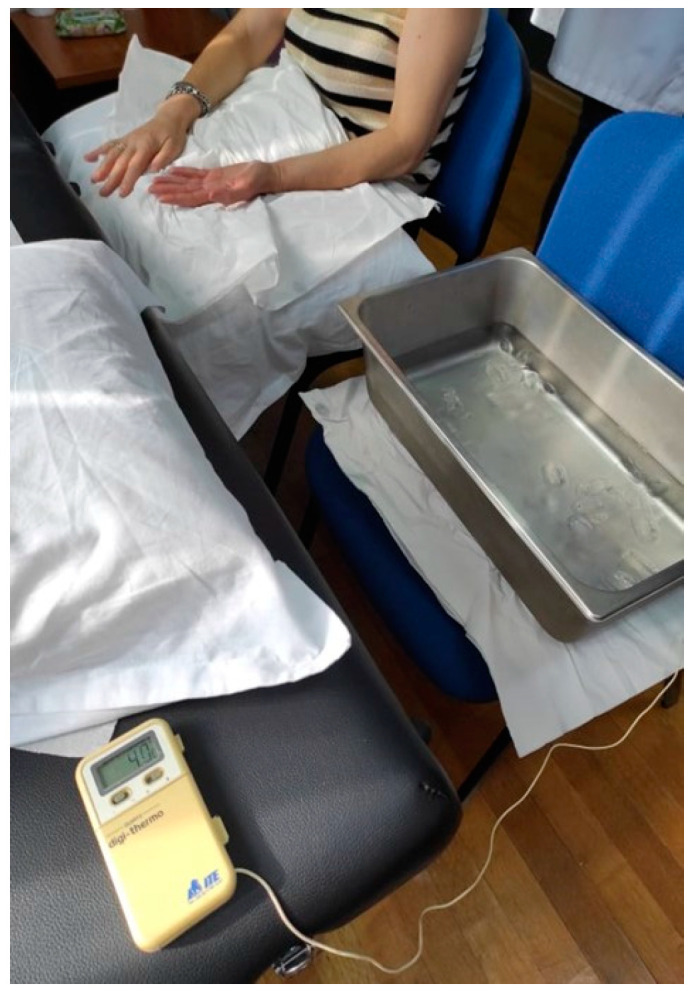
CPM procedure.

In order to assess CPM, PPT was used as a tested stimulus on the dominant or painful shoulder in the upper trapezius muscle [30]. Baseline PPTs were assessed three times with an interval of 30 s between the repetitions. A Cold Pressure Task (CPT) was used as the conditioning stimulus. We chose the specific combination of these two stimuli for our experiment based on the existing literature, which indicates that this particular pairing (PPT and CPT) has shown higher reliability [43]. Participants were instructed to immerse their non-dominant or uninvolved hand in the ice water bath for 20 s twice with a 2 min rest period between each conditioning stimulus [44] (Figure 3). 

**Figure 4:**
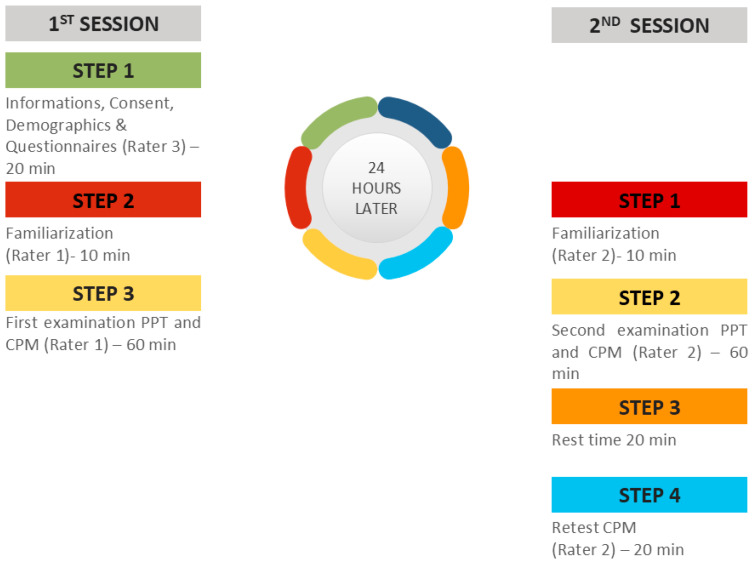
Study design of the experimental sessions.

When the participants removed the hand from the water, they were instructed to rest it on a soft towel without rubbing it so as not to receive strong stimuli or a sudden increase in the temperature of the hand. The water was maintained at a constant temperature of 4 °C [44]. Two minutes after the immersion of the hand, a new PPT was delivered. After a 20 min rest period, the CPM procedure was repeated by the same rater in order to assess the intra-rater reliability (Figure 4). A CPM index was calculated as the percent ratio of the mean PPTs after the CPTs (after the immersion) to PPTs before CPTs. 

### 2.6. Self-Reported Outcome Measures

The self-reported outcome measures used in this study were Greek versions of the International Physical Activity Questionnaire, the Hospital Anxiety and Depression Scale, and the Central Sensitization Inventory. 

The short form of the International Physical Activity Questionnaire (IPAQ) was used to estimate the level of physical activity of the participants. By completing the IPAQ, the respondent is asked to answer 7 questions concerning activities of 4 intensity levels (vigorous activity, moderate-intensity activity, walking, and sitting) performed in the last week [45]. The IPAQ was adapted culturally into Greek and the reliability of the Greek version of IPAQ was acceptable [46]. 

The Hospital Anxiety and Depression Scale (HADS) was used as a measure of anxious and depressive symptomatology [47]. The HADS consists of 14 items describing various symptoms of anxiety and depression. Seven items refer to anxiety and seven to depression. Responses are summed up to yield an overall score for each category. Previous studies have shown that the Greek version of HADS is a reliable tool [48]. 

The Central Sensitization Inventory (CSI) is a self-reported screening instrument that assesses the symptoms related to CS. The CSI consists of two parts. Part A includes 25 statements measured on a 5-point Likert scale (0 = never, 4 = always) [49]. A cut-off score ≥ 40/100 indicates the presence of CS. The Greek version of CSI has excellent reliability in patients with chronic pain [50]. 

### 2.7. Before the Testing Procedure

Patients communicated with the researchers by phone. The researchers obtained a brief history and checked the selection and exclusion criteria. If the volunteers met the selection criteria, the dates of the sessions were set. Two sessions were necessary to assess the reliability. Each session lasted 1 h and 30 min. Also, the researchers informed the volunteers about the procedure and the necessary clothing they had to wear during the experimental sessions and instructed them to avoid vigorous physical activity, alcohol, nicotine, caffeine, and analgesics consumption. 

### 2.8. During the Testing Procedure

In the first session, a researcher (PB) welcomed, informed, and obtained the demographic characteristics (age, sex, height, weight, occupation, and level of education) of the subjects. All the participants were informed and gave their written consent before their participation. Examinees were free to ask questions about the tests and the purpose of the study. Participants also completed questionnaires on physical activity, psychological distress, and symptoms related to CS. Then, the first rater (PKM) performed the two tests (PPT and CPM) in random order. The second rater (NV) repeated the same procedure 24 h later. During the measurements, the temperature, lighting, and humidity of the rooms were kept constant.

### 2.9. Statistical Analysis

The Statistical Package for Social Sciences (SPSS) version 23.0 was used for the analysis. Data collected checked for normality with the Kolmogorov–Smirnov test and descriptive statistics were conducted for all variables. The reliability analysis was performed by using the ICC for the total sample and subgroup reliability analysis for patients and healthy volunteers, Standard Error of Measurement (SEM = √σerror) and Smallest Detectable Change (SDC = 1.96 × √2 × SEM) were calculated. We selected ICC (2,1), which is a two-way random effects model, to assess inter-rater reliability because it is an appropriate model for evaluating absolute agreement between raters when the goal is to generalize the findings to a broader population of raters with similar characteristics to those in our study. Additionally, since our analysis was based on measurements obtained by a single rater at a time, the “single rater” type was deemed appropriate. For the assessment of intra-rater reliability, we selected the two-way mixed-effects, absolute agreement, single rater/measurement form of ICC. The two-way mixed-effects model is appropriate for testing intra-rater reliability with multiple scores from the same rater, as it is not intended to generalize one rater’s scores to a larger population of raters. This model is also suitable for test–retest reliability studies because repeated measurements by the same rater cannot be regarded as randomized samples. Additionally, we used this model to assess the absolute agreement between repeated measures, which was critical for our study’s objectives [51]. The ICC ranges from 0 to 1, with values under 0.5 reflecting poor reliability, values between 0.5 and 0.75 indicating moderate reliability, values from 0.75 to 0.9 representing good reliability, and values above 0.9 signifying excellent reliability [51].

The PPTs between patients and healthy subjects were compared by using an independent *t*-test. 

## 3. Results

### 3.1. Descriptive Statistics

A convenience sample of 51 participants (30 males/21 females) participated in the study. The mean age of the subjects was 30.79 ± 13.1 years, with a range from 19 to 63 years and the mean BMI was 23.7 ± 4.02 kg/m^2^. Most participants (80.8%) had the right upper extremity as dominant and 42.3% had at least a bachelor’s degree. The total sample was stratified into symptomatic (n = 20) and asymptomatic participants (n = 31). The groups’ profiles are summarized in Table 1. 

There was no statistically significant difference between groups in age, BMI, and fitness level. Patients showed statistically higher scores of pain intensity, pain duration, anxiety, depression, and symptoms related to central sensitization.

### 3.2. Pressure Pain Threshold 

#### Intra-Rater and Inter-Rater Reliability

Table 2 presents the, ICC, SEM, and SDC values of PPT for the asymptomatic (n = 31) and the symptomatic groups (n = 20). The intra-rater reliability was similar between the two groups (asymptomatic and symptomatic participants) (Table 2). According to the results, the intra-rater reliability (ICC = 0.96–0.99, 95% CI = 0.91–0.998) was excellent for PPT. The SEM ranged between 22.93 and 44.76 kPa and the SDC was calculated at 18.84–42.36.

The inter-rater reliability is generally high in the asymptomatic group, with ICC values ranging from 0.916 to 0.958, indicating excellent reliability. In contrast, the symptomatic group shows a wider range of ICC values (0.59 to 0.89), suggesting variability in inter-rater reliability, with some measurements showing lower reliability.

In the asymptomatic group, SEM values range from 49.61 to 61.41 (except for PPT4, SEM = 103.12) kPa. These relatively lower SEM values indicate that the measurements are fairly precise and consistent among different raters, which is essential for reliable assessment in clinical settings. Conversely, in the symptomatic group, SEM values are higher, ranging from 73.83 to 121.98 kPa. Higher SEM values suggest less precision in the measurements when different raters assess symptomatic individuals.

Nevertheless, the asymptomatic group presented higher inter-rater reliability while poor reliability was found for the examined site 1 (deltoid muscle) in the symptomatic group (Table 2). 

### 3.3. Conditioned Pain Modulation 

#### Intra-Rater and Inter-Rater Reliability 

Table 3 presents the intra-rater and inter-rater reliability of CPM) for asymptomatic and symptomatic groups. The reliability of CPM in the symptomatic group was higher than in healthy participants when the test was conducted on the same day by the same rater and extremely low when different raters carried out it in patients (Table 3). 

For intra-rater reliability, the symptomatic group showed higher reliability with an ICC of 0.816 compared to the asymptomatic group’s ICC of 0.669. The SEM and SDC were also lower in the symptomatic group (8.314 kPa and 20.696 kPa) than in the asymptomatic group (13.393 kPa and 31.393 kPa), indicating more precise and detectable changes in symptomatic patients.

For inter-rater reliability, both groups showed low ICC values, with the symptomatic group at 0.074 and the asymptomatic group at 0.365, indicating poor agreement between different raters. The SEM and SDC were considerably higher in both groups, particularly in the symptomatic group (25.364 kPa and 63.526 kPa), compared to the asymptomatic group (20.295 kPa and 47.519 kPa). This suggests greater measurement error and larger detectable changes needed for reliability in inter-rater assessments. 

### 3.4. Correlations between Self-Reported Questionnaires and PPTs 

A statistically significant negative but weak correlation was found between PPT3 (levator scapula) and CSI (r = −0.29, *p* = 0.020) in the entire sample. However, no statistically significant correlation was observed between PPTs at different points and the other self-reported questionnaires. Within the patient sample, no statistically significant correlation was identified between the PPTs at different points and all self-reported questionnaires. Statistically significant negative weak correlations were discovered between PPT1 (trapezius) r = −0.381, *p* = 0.041, PPT2 (deltoid) r = −0.415, *p* = 0.025, PPT3 (levator scapulae) r = −0.429, *p* = 0.020, as well as PPT4 (tibialis) r = −0.389, *p* = 0.040 and CSI in healthy individuals.

### 3.5. Correlations between Self-Reported Questionnaires and CPM 

The total sample did not reveal any statistically significant correlation between the CPM index and the self-reported questionnaires. Within the healthy sample, a statistically significant negative but weak correlation was observed between the CPM index and anxiety (r = −0.379, *p* = 0.043) as well as HADS total score (r = −0.449, *p* = 0.014). Among the patient sample, a statistically significant negative and moderate correlation was identified between the CPM index and the IPAQ total score (r = −0.550, *p* = 0.012).

## 4. Discussion

To the best of our knowledge, this is the first study to investigate both the intra-rater and inter-rater reliability of PPT and CPM in the same sample of patients with chronic shoulder pain and healthy participants. According to the results of the present study, PPT proved to be a reliable outcome measure. However, the inter-rater reliability was moderate to good in the symptomatic group. On the other hand, moderate intra-rater and poor inter-rater reliability were found for CPM. The symptomatic group had higher test–retest reliability than the control group for CPM, but it presented extremely low inter-rater reliability. 

### 4.1. Pressure Pain Threshold

The reliability of PPT in the shoulder has previously been examined in healthy subjects showing that PPT is a reliable measurement [52,53,54,55]. These results are consistent with the results of the present study. Nevertheless, the information extracted from the PPT is particularly useful in populations with painful disorders such as shoulder pain, as discussed in the introduction. Only two studies used patients with shoulder pain [56]. Vandeweeën et al. [56] evaluated the intra-rater reliability of PPT in 30 patients with unilateral shoulder and arm pain of at least 2 months’ duration. The examined sites evaluated were trigger points, eight paravertebral and six in the shoulder and arm. The intra-rater reliability was found to be moderate to excellent but there is no information on which points showed greater reliability. It is reported that the painful area had a higher reliability index than the non-painful area. Nascimento et al. [30] conducted an inter-rater and intra-rater reliability study in participants with and without subacromial impingement syndrome and found good to excellent reliability. The muscles evaluated were the lower and upper trapezius, the infraspinatus and the medial deltoid in contrast to our study in which the upper trapezius, levator scapulae, and medial deltoid were assessed. Comparing the results in the present study, the inter-rater reliability in the patient group was lower for both upper trapezius and medial deltoid muscles. This difference can be attributed to two main factors. Initially, in the study by Nascimento et al. [30], the measurements were performed on the same day, while in the current study, the measurements were performed over a 24 h period. Also, the heterogeneity of the sample may be responsible for the differences in the results. We recruited patients with chronic shoulder pain while Nascimento et al. included patients with subacromial impingement syndrome and pain for at least 1 month. The intra-rater reliability was similar for the two studies. 

In this study, SEM and SDC which are useful in interpreting PPT changes in treatment, were calculated. In general, SEM in intra-rater measures was found 25.21–44.75 kPa for the asymptomatic group and 22.93–33.58 kPa for the patients. Although the study by Nascimento et al. [30] used different units, knowing that 1 kgf/cm^2^ equals 98.07 kPa, we conclude that the present study exhibits a lower measurement error. However, inter-rater measures for asymptomatic but mainly for the group of patients showed SEM values with extremely high variability. Previous studies have reported that the rate of pressure and the examiner’s reaction time when the examinee says “stop” can affect the results. However, this is not correct in the present study as a switch was used and the selected algometer includes visual feedback to the examiner for the correct pressure rate. Although, in the intra-rater measures, a change of approximately 18–25 kPa is enough to be deemed meaningful, in the inter-rater measures, change must be between 42.01–56.28 kPa for the healthy participants and 61.57–97.58 kPa for the patients. The findings show that clinicians would need to detect a change of 61–98 kPa to be confident that PPT presents a true change. The diurnal variation could account for the variability; however, given that the participants included in the analysis reported adherence to the instructions about physical activity and caffeine and analgesics consumption and did not report changes in stress or mood levels over 24 h, questions are raised about the reliability of the measure and its use in clinical practice. Nascimento et al. [30] pointed out that each examiner should know his absolute reliability concerning the population under study.

### 4.2. Conditioned Pain Modulation 

A variety of stimuli are used as conditioning or test stimuli in order to assess the CPM. In this study, PPT was used as a tested stimulus and CPT as a conditioning stimulus because the combination of the two stimuli presented higher intra-rater reliability in patients with chronic pain [43]. The subgroup analysis revealed that the intra-rater reliability of CPM was good in patients and moderate in the asymptomatic group, but the inter-rater reliability was poor, especially in patients. 

According to a recent systematic review [32], four studies investigated the reliability of CPM in the region of the shoulder [28,33,34,35] but only one involved recruited patients with shoulder pain (acute or subacute stage) [28]. More specifically, the stability of CPM was investigated between women and men. The results showed that the intraday intra-rater reliability was moderate for women (ICC = 0.65) and fair for men (ICC = 0.40) in the symptomatic group. The interday intra-rater reliability was moderate (ICC = 0.59–0.61) for both men and women [28]. 

Several studies have attempted to explain what factors can affect the repeatability of CPM. Mood [57], distraction [57], menstrual phase [58], catastrophic thoughts [59], physical activity, and the examined site [60] can partially influence the activity of the endogenous pain inhibitory system. However, even these factors cannot explain the large variability in the measurements [61]. Possible inter-individual factors affect CPM. Since not the entire population is expected to show similar results on the CPM, comparisons of participants’ means are likely to add more confounding factors to researchers. More specifically, it is possible that even in healthy people, CPM can show a facilitatory effect. It has been proposed to classify participants according to their response to the CPM in order to draw safer conclusions [62].

### 4.3. Self-Reported Questionnaires’ Associations with PPTs and CPM

In the entire sample, there was a statistically significant negative and weak correlation observed between PPT3 (levator scapula) and the CSI score, indicating that as the CSI score increased, the PPT3 decreased. Within the patient sample, no significant correlations were found between PPTs at different points and the self-reported questionnaires. Our findings are consistent with a previous study that reported no significant association between the CSI and the PPTs in patients with chronic unilateral shoulder pain [63]. In contrast, in healthy individuals, a statistically significant negative correlation was found between PPTs at various points (trapezius, deltoid, levator scapulae, and tibialis) and the CSI score, indicating that as the CSI score increased, the PPTs at these points decreased. We hypothesize that this association can be explained, in part, by the fact that the healthy sample had a higher mean depression score than the patients, although the mean was low (3.06 ± 2.65). We cannot declare that the healthy subjects had increased symptoms of depression, but it is possible that this difference between the two groups affected the association with CSI. The CSI encompassed a wide range of symptomatology related to sensitization and not solely restricted to alterations in nociceptive pain processing [64]. The items within the CSI measure a variety of constructs such as physical, psychological, and cognitive functioning, as well as physical symptoms among others. These constructs are likely associated with CS, but additional research is necessary to confirm the CSI’s validity as a measure for CS [65].

There was a significant negative correlation between two measures, the CPM index and anxiety, as well as HADS total score. The CPM is a process by which pain sensitivity is inhibited by pain-suppressing mechanisms in the nervous system. A higher CPM index indicates that an individual has better pain-inhibiting capacity [66]. Anxiety is a psychological construct that relates to the feelings of apprehension and fearfulness experienced by an individual [67]. HADS is a questionnaire that measures the severity of anxiety and depression symptoms [47]. The negative correlation observed between the CPM index and anxiety/HADS total score implies that individuals with higher anxiety or more severe anxiety/depression symptoms tend to have a lower CPM index, which means they have a reduced ability to inhibit pain perception. This finding may indicate a link between psychological states and pain sensitivity in healthy individuals. On the other hand, the negative and moderate correlation observed between the CPM index and the IPAQ total score means that patients who are more physically active tend to have a higher CPM index, implying that they have better pain-inhibiting capacity. The results suggest that physical activity may have a beneficial effect on pain inhibition in patients, and therefore, it could be a helpful complementary therapy in pain management. Previous studies support that both older and younger adults who were less sedentary and had a higher level of light physical activity per day demonstrated greater pain inhibitory capacity [68,69].

### 4.4. Limitations and Strengths

Some important limitations should be addressed by future research in order to strengthen the results of the study. Initially, inter-examiner measurements were performed at 24 h intervals. Measuring on a different day could partially affect the stability of the measurements. The decision to space measurements was also intended to mitigate the potential for measurement area irritation or sensitization due to repeated or immediate assessments. This approach was adopted to evaluate the stability of PPT measurements under conditions that simulate a more realistic clinical setting, where time lapses between assessments are common. To check the stability of the subjects’ condition, the tests were performed at the same time, clear instructions were given regarding physical activity, sleep, consumption of analgesics, alcohol, and caffeine, and the patients’ self-reported anxiety and mood were assessed before the two measurements. Also, the women evaluated were not menstruating. Second, although each rater was blind to the other’s results, he was not blind to his own results. That is, the raters knew the repeated measurements of each point. This limitation was partially mitigated as measurements of one point were not repeated but evaluated cyclically after measuring other points. So, the rater would have a hard time remembering the performance of the first point after the other iterations. Also, the third rater entered the scores into the evaluation booklet. Thirdly, patients could be separated according to pain intensity to extract more information about the activation of the endogenous pain inhibitory system; however, the sample was smaller than the estimated sample and the analyses would not have power, but also a previous study showed that variations in pain intensity did not affect CPM [28]. The mean and standard deviation for the water temperature during the test procedures were not recorded, which is recognized as a limitation of the study. Finally, the results of the study can be used to conclude the specific population studied and the use of specific stimuli when conducting the CPM. Despite these limitations, the present study is a novel contribution to the literature by evaluating the stability of PPT and CPM in patients with chronic shoulder pain. Controlling for factors such as patient stability (stress, menstrual phase, mood, sleep, physical activity, alcohol, caffeine, and analgesic consumption), stability of environmental conditions (temperature, humidity, lighting), rater training, blinding to the assessment of inter-examiner reliability, and randomization of the tests helped to strengthen the results of this study. 

### 4.5. Implications in Clinical Practice

The study confirms that PPT is a reliable outcome measure, especially for intra-rater assessments in patients with chronic shoulder pain. This reliability supports the use of PPT in clinical practice for monitoring changes in pain sensitivity over time. Given that PPT measurements showed lower inter-rater reliability in symptomatic patients compared to asymptomatic individuals, clinicians should be cautious when interpreting results across different examiners. Standardizing measurement techniques and providing thorough training for clinicians can help mitigate some of the variability observed in this study. Additionally, understanding the expected SEM and SDC values allows clinicians to discern whether changes in PPT are clinically meaningful, ensuring that treatment decisions are based on reliable data.

The study’s results highlight the variability and challenges associated with CPM measurements, particularly in terms of inter-rater reliability. The moderate intra-rater reliability observed in both symptomatic and asymptomatic groups suggests that CPM can be a useful tool for assessing pain modulation in a controlled setting. However, the poor inter-rater reliability, especially in symptomatic patients, indicates that CPM should be interpreted with caution when used in clinical practice. This variability may stem from individual differences in pain processing or other external factors, emphasizing the need for clinicians to be aware of the limitations of CPM as a diagnostic tool. Nevertheless, the association between higher physical activity levels and better pain inhibitory capacity, as measured by CPM, suggests that promoting physical activity could be beneficial in managing pain in patients with chronic shoulder pain.

## 5. Conclusions

This study is the first to evaluate both intra-rater and inter-rater reliability of the Pressure Pain Threshold (PPT) and Conditioned Pain Modulation (CPM) in patients with chronic shoulder pain and healthy participants. The findings indicate that the PPT is a reliable outcome measure, particularly for intra-rater assessments in patients with chronic shoulder pain, supporting its use in clinical practice for monitoring pain sensitivity. However, the lower inter-rater reliability observed in symptomatic patients highlights the need for standardized measurement techniques and thorough training of clinicians to reduce variability.

On the other hand, the CPM exhibited moderate intra-rater reliability but poor inter-rater reliability, especially in the symptomatic group. This variability suggests that while CPM can be useful for assessing pain modulation in a controlled setting, its application in clinical practice should be approached with caution. The study also found that higher levels of physical activity were associated with better pain inhibitory capacity, suggesting that promoting physical activity could be a beneficial strategy for managing pain in patients with chronic shoulder pain.

Overall, while the PPT shows promise as a reliable tool for assessing pain sensitivity, the limitations associated with CPM’s reliability should be considered when incorporating these measures into clinical practice.

## Figures and Tables

**Table 1:** Characteristics of the symptomatic and asymptomatic group.

Variable	Asymptomatic Group (N = 31)	Symptomatic Group (N = 20)	*p*-Value
Age	28.88 (11.556)	33.850 (15.045)	0.312
Height	1.717 (0.096)	1.752 (0.108)	0.291
Weight	70.30 (14.554)	71.825 (13.889)	0.618
BMI	23.633 (3.209)	23.792 (5.147)	0.880
HADS	7.370 (4.682)	7.400 (4.661)	0.962
HADS Anxiety	4.310 (2.845)	4.775 (3.427)	0.043 *
HADS Depression	3.060 (2.651)	2.800 (2.563)	0.025 *
CSI	22.720 (17.139)	27.550 (8.835)	0.027 *
IPAQ	1796.99 (1528.934)	1150.875 (601.350)	0.185
IPAQ Vigorous Score	721.250 (1103.729)	282.800 (286.438)	0.238
IPAQ Moderate Score	551.250 (907.132)	265.000 (220.132)	0.354
IPAQ Walking Score	524.490 (584.846)	603.075 (380.743)	0.113

* statistically significant difference.

**Table 2:** Intra-rater and inter-rater reliability of PPT for the asymptomatic (n = 31) and the symptomatic groups (n = 20).

	Points	Intra-Rater Reliability		Inter-Rater Reliability	
		Asymptomatic Group	Symptomatic Group	Asymptomatic Group	Symptomatic Group
ICC (95% CI)	PPT1	0.95 (0.91–0.97)	0.96 (0.91–0.98)	0.936 (0.867–0.969)	0.59 (0.435–0.80)
	PPT2	0.98 (0.961–0.99)	0.97 (0.94–0.99)	0.953 (0.904–0.977)	0.83 (0.587–0.90)
	PPT3	0.96 (0.93–0.98)	0.96 (0.93–0.98)	0.916 (0.828–0.960)	0.77 (0.431–0.91)
	PPT4	0.99 (0.98–0.99)	0.98 (0.95–0.99)	0.958 (0.915–0.980)	0.89 (0.72–0.95)
SEM (kPa)	PPT1	44.76	26.29	61.41	121.98
	PPT2	28.16	27.61	54.70	90.22
	PPT3	25.21	22.93	49.61	73.83
	PPT4	33.27	33.59	103.12	103.04
SDC (kPa)	PPT1	42.36	25.13	52.22	97.59
	PPT2	22.52	22.30	42.01	67.35
	PPT3	25.96	23.60	48.03	65.16
	PPT4	18.84	20.48	56.29	61.58

PPT1 = PPT in the middle deltoid, PPT2 = PPT in the levator scapulae, PPT3 = PPT in the upper trapezius, PPT4 = PPT in the belly of the tibialis anterior muscle, ICC = Intraclass Correlation Coefficient, SEM = Standard Error of Measurement, SDC = Smallest Detectable Change.

**Table 3:** Intra-rater and inter-rater reliability of CPM for the asymptomatic (n = 31) and symptomatic (n = 20) groups.

**Intra-Rater Reliability**							
		**CPM Index**	**CPM Index (Retest)**				
	n	Mean (SD)	Mean (SD)	ICC	95% CI	SEM (kPa)	SDC (kPa)
Symptomatic group	20	112.044 (14.683)	110.652 (14.697)	0.816	0.544–0.927	8.314	20.696
Asymptomatic group	31	120.908 (20.989)	115.599 (17.494)	0.669	0.319–0.840	13.393	31.393
**Inter-Rater Reliability**							
		**CPM Index (Rater 1)**	**CPM Index (Rater 2)**				
		Mean (SD)	Mean (SD)	ICC	95% CI	SEM	SDC
Symptomatic group	20	112.044 (14.683)	109.293 (33.041)	0.074	−1.299–0.631	25.364	63.526
Asymptomatic group	31	120.908 (20.989)	115.856 (24.875)	0.365	−0.307–0.692	20.295	47.519

CPM = Continued Pain Modulation, SD = standard deviation, ICC = Intraclass Correlation Coefficient, SEM = Standard Error of Measurement, SDC = Smallest Detectable Change.

## Data Availability

The data provided in this research can be obtained upon request from the corresponding author.
